# A Non-Classical Presentation of Erythema Migrans in a 51-Year-Old Woman With Early Manifestation of Lyme Neuroborreliosis (Bannwarth Syndrome)

**DOI:** 10.7759/cureus.39931

**Published:** 2023-06-04

**Authors:** Jovans R Lorquet, Robert Pell, Jeffrey Adams, Mihir Tak, Latha Ganti

**Affiliations:** 1 Emergency Medicine, HCA Florida Osceola Hospital, Kissimmee, USA; 2 Emergency Medicine, Envision Physician Services, Plantation, USA; 3 Emergency Medicine, University of Central Florida College of Medicine, Orlando, USA

**Keywords:** bannwarth syndrome, borrelia burgdorferi, erythema chronicum migrans, lyme neuroborreliosis, lyme disease

## Abstract

The authors present a case of a 51-year-old female who presented to the emergency department with general malaise, headache, neck stiffness, and an expanding rash consistent with Lyme neuroborreliosis. In this case report, the clinical presentation, diagnosis, and management of Lyme neuroborreliosis and different presentations of erythema migrans are discussed.

## Introduction

Defining Lyme disease

Lyme disease is the most commonly encountered vector-borne disease in the United States. It is estimated that there are nearly half a million cases per year [1]. Lyme disease is most commonly caused by *Borrelia burgdorferi*, a spirochetal bacterium that can be transmitted to human hosts via tick vectors, which most commonly belong to the *Ixodes *species [1]. Lyme disease affects multiple systems, most commonly the skin, followed by the nervous system. Other possible but less common manifestations include carditis, keratitis, uveitis, and inflammatory arthritis [2].

Characteristic “bull's-eye” rash

One of the most common and well-known skin manifestations of Lyme disease is the classic bull's-eye rash or “erythema migrans.” Unfortunately, it may be difficult to diagnose clinically as it may present in a multitude of ways and may mimic other disease processes, such as cellulitis and various insect bites. A variation of this rash will be explored in this case report.

Stages of Lyme disease

Lyme disease is divided into stages, and there is an acute localized stage (erythema migrans) and a disseminated stage, which in turn is divided into early and late phases. Neurological symptoms tend to occur during the disseminated stage, and both the central nervous system (CNS) and peripheral nervous system (PNS) can be affected. CNS involvement is rare in the acute stage of Lyme disease [3].

Lyme neuroborreliosis (LNB)

LNB also named Bannwarth syndrome in Europe is simply defined as neurologic involvement during the disseminated stage of Lyme disease/*B. burgdorferi *infection. Common features of LNB include cerebrospinal fluid (CSF) pleocytosis that is primarily lymphocytic in nature, aseptic meningitis, radiculitis, cranial neuritis, cranial nerve VII palsy, and symptoms occurring weeks to months after the initial infection [4]. To make a diagnosis, there has to be laboratory findings consistent with Lyme disease through western blot/serology, and there must be clinical signs of the disease with a history of erythema migrans/rash. Some researchers report that meningitis is the most common single, early manifestation of LNB/Bannwarth syndrome in North America [3].

## Case presentation

A 51-year-old female with a past medical history significant for Roux-en-Y gastric bypass presented to the emergency department with four days of worsening headache, nausea, malaise, and extensive rash on her back. The patient stated that she started to feel unwell and noticed a new large rash developing on her upper back while she was in her hometown in Wisconsin. She stated that it started as a small area of redness, spreading rapidly. She could not recall any history of insect bites, had not started any new medications, and had no exposure to known allergens. The patient was seen in the emergency department and prescribed cephalexin and valacyclovir due to suspicion of cellulitis versus herpes zoster at the time. Over the course of the next week, she reported steadily feeling worse. She stated that the rash had gotten larger and more pruritic and that her headache had become more severe, also causing severe pain that radiated to the right side of her neck. The patient denied fever, chest pain, shortness of breath, trauma, immunocompromised status, intravenous drug use, steroid use, sudden onset headache, numbness, focal weakness, or tingling of extremities.

Vital signs were within normal limits with an oral temperature of 36.8^o^C, pulse of 90 beats per minute, respiratory rate of 19, and blood pressure of 130/75 mmHg. A physical exam revealed the presence of a large erythematous rash covering two-thirds of the right side of the back, with a crusted plaque located centrally, measuring approximately 5 cm in diameter. There was also an area of mild erythema in the right postauricular area. The neurological exam did not reveal focal weakness except for intense radiating neck pain with rotation. Cardiac, pulmonary, and all other systems were otherwise unremarkable. Symptomatic treatment was initiated with intravenous ondansetron, ketorolac, pantoprazole, and normal saline. Oral doxycycline hyclate (100 mg) was added for the empiric treatment of Lyme disease, which was added to the differential at the time. Tests for Lyme serology and western blot were sent out.

**Figure 1:**
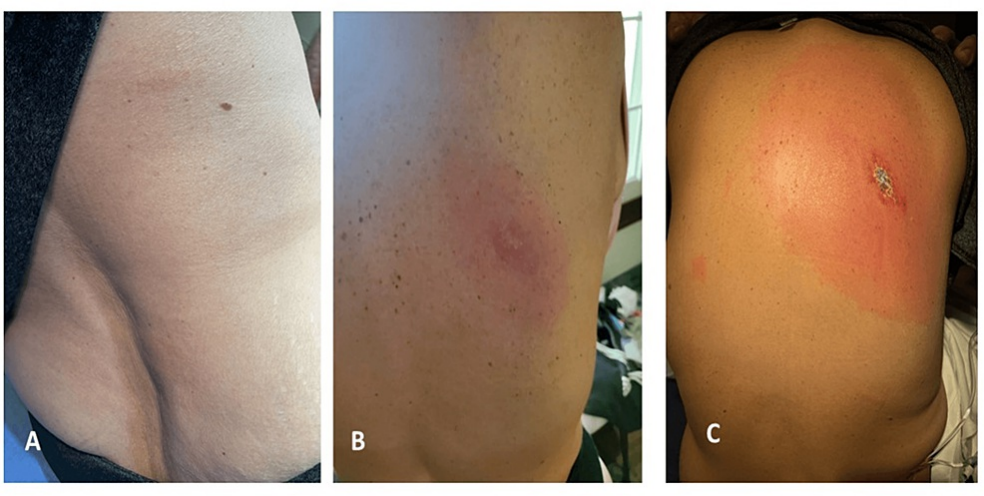
Progression of Lyme lesion on the patient's back

The patient had documented the progression of her rash (Figure 1).

**Figure 2:**
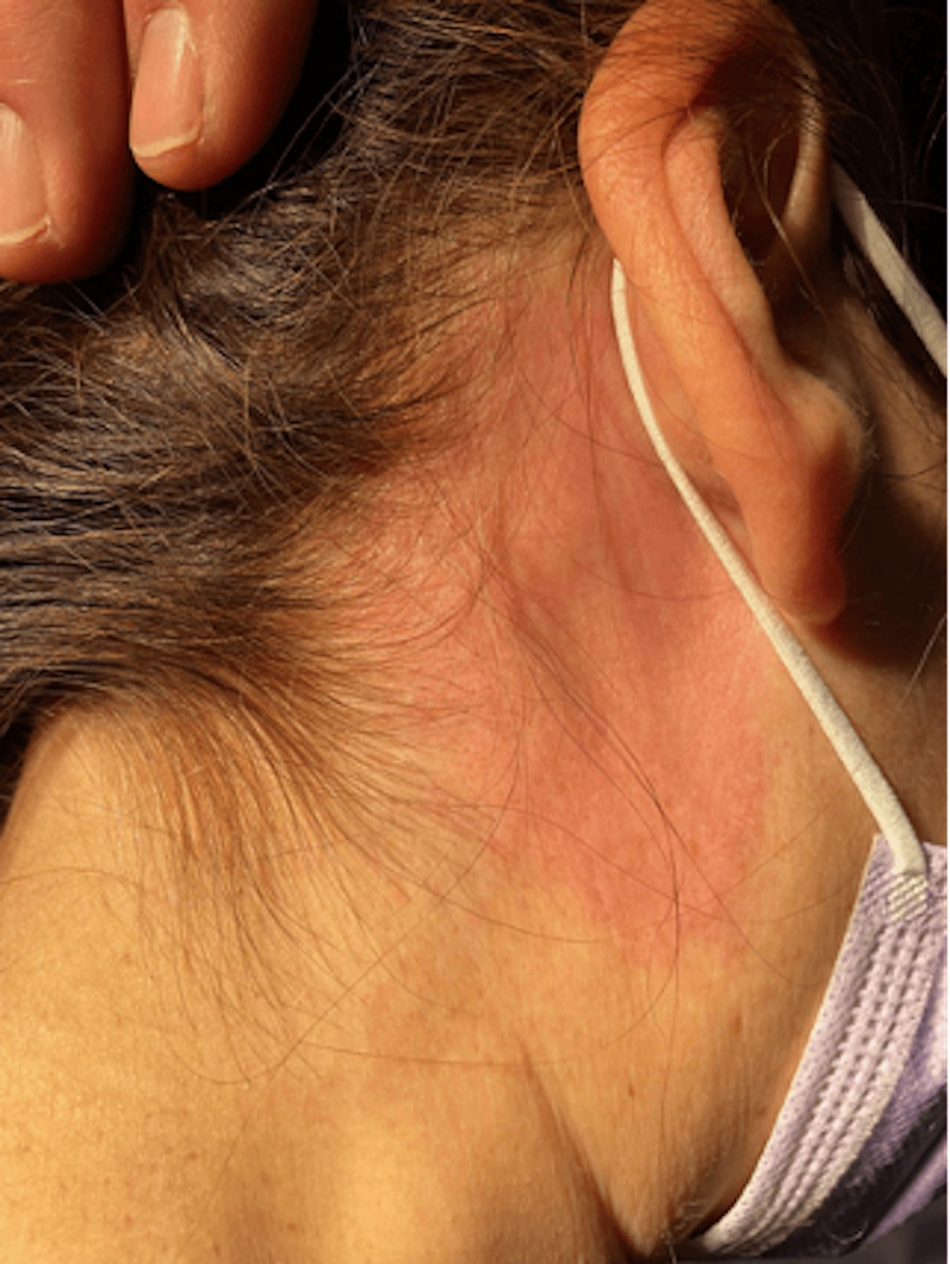
Right postauricular erythema

It can be seen that the cutaneous manifestation of the disease had remained different from the classical depiction of erythema migrans, regardless of its stage. In addition, signs of dissemination in the form of a right postauricular erythema were also present (Figure 2). On a follow-up several weeks later, the patient had made a full recovery and was back to her baseline level of functioning. 

## Discussion

Early disseminated infection with *B. burgdorferi *affecting the CNS (within 2-10 weeks of the tick bite) has been referred to as LNB or Bannwarth syndrome in Europe [5,6]. Although this syndrome refers more to severe, migrating radicular pain that can be accompanied by peripheral nerve paresis in the context of erythema migrans, LNB can also present often with uni- or bilateral facial palsy and sometimes CSF pleocytosis as seen in our patient. Of note, it is reported that lymphocytic meningitis is the most common single, early manifestation of LNB/Bannwarth syndrome in North America [3]. Thus, based on the lymphocytic meningitis findings, LNB is high on the differential. Our patient met the CSF criteria for lymphocytic meningitis, and based on the atypical erythema migrans findings, positive Lyme IgM, and roughly two weeks of symptoms with worsening severe headache and neck radiculopathy, the diagnosis of LNB can be made. Our patient had disseminated (stage 2) Lyme disease, evidenced by the presence of the main extensive rash on the back and additional rash in the right postauricular area.

## Conclusions

The “bull's-eye” appearance of erythema migrans is not the only cutaneous manifestation of the acute stage of Lyme disease. There can be multiple variations of the rash, as demonstrated in the patient. A high level of clinical suspicion combined with serology, western blot testing, and empiric treatment provides the most optimal management of the consequences of *B. burgdorferi *infection. LNB, also named Bannwarth syndrome, presents with neurologic symptoms in the context of disseminated Lyme disease. Findings such as rash following a tick bite, headache symptoms, radiculitis, aseptic meningitis, and CSF lymphocytosis, in association with positive Lyme IgM/IgG, should allow physicians to make the diagnosis confidently.
